# Further insight into the geographic distribution of *Leishmania* species in Peru by cytochrome *b* and mannose phosphate isomerase gene analyses

**DOI:** 10.1371/journal.pntd.0007496

**Published:** 2019-06-20

**Authors:** Hirotomo Kato, Abraham G. Cáceres, Chisato Seki, Carmen Rosa Silupu García, Carlos Holguín Mauricci, Salvadora Concepción Castro Martínez, Dafne Moreno Paico, Josefa Leila Castro Muniz, Lucinda Doriz Troyes Rivera, Zoila Isabel Villegas Briones, Silvia Guerrero Quincho, Guísela Lucy Sulca Jayo, Edwin Tineo Villafuerte, Carlos Manrique de Lara Estrada, Fernando Rafael Arias, Fredy Santiago Passara, Nancy Ruelas Llerena, Makoto Kubo, Ahmed Tabbabi, Daisuke S. Yamamoto, Yoshihisa Hashiguchi

**Affiliations:** 1 Division of Medical Zoology, Department of Infection and Immunity, Jichi Medical University, Tochigi, Japan; 2 Sección de Entomología, Instituto de Medicina Tropical “Daniel A. Carrión” y Departamento Académico de Microbiología Médica, Facultad de Medicina Humana, Universidad Nacional Mayor de San Marcos, Lima, Perúu; 3 Laboratorio de Entomología, Instituto Nacional de Salud, Lima, Perúu; 4 Laboratorio de Parasitología, Dirección de Laboratorio de Salud Pública, Dirección Regional de Salud Piura, Piura, Peru; 5 Laboratorio del Centro de Salud Motupe, Micro Red de Salud Motupe, Red de Salud Lambayeque, Gerencia Regional de Salud Lambayeque, Lambayeque, Peru; 6 Laboratorio del Comité Local de Administración en Salud (CLAS) de Colasay, Sub Región de Salud Jaén, Dirección Regional de Salud Cajamarca, Cajamarca, Peru; 7 Laboratorio de Referencia Regional de Salud Pública, Dirección Regional de Salud Ayacucho, Ayacucho, Peru; 8 Laboratorio de Referencial Regional de Salud Púbica, Dirección Regional de Salud Madre de Dios, Madre de Dios, Peru; 9 Laboratorio de Referencia Regional de Salud Pública, Dirección Regional de Salud Puno, Puno, Peru; 10 Departamento de Microbiología y Patología, Facultad de Medicina, Universidad Nacional de San Agustín, Arequipa, Peru; 11 Division of Immunology, Kitasato University School of Allied Health Sciences, Kanagawa, Japan; 12 Departamento de Parasitologia y Medicina Tropical, Facultad de Ciencias Medicas, Universidad Catolica de Santiago de Guayaquil, Guayaquil, Ecuador; Centro de Pesquisa Gonçalo Moniz-FIOCRUZ/BA, BRAZIL

## Abstract

To obtain further insight into geographic distribution of *Leishmania* species in Peru, a countrywide survey, including central to southern rainforest areas where information on causative parasite species is limited, was performed based on cytochrome *b* (*cyt* b) and mannose phosphate isomerase (*mpi*) gene analyses. A total of 262 clinical samples were collected from patients suspected of cutaneous leishmaniasis (CL) in 28 provinces of 13 departments, of which 99 samples were impregnated on FTA (Flinders Technology Associates) cards and 163 samples were Giemsa-stained smears. *Leishmania* species were successfully identified in 83 (83.8%) of FTA-spotted samples and 59 (36.2%) of Giemsa-stained smear samples. Among the 142 samples identified, the most dominant species was *Leishmania (Viannia) braziliensis* (47.2%), followed by *L*. *(V*.*) peruviana* (26.1%), and others were *L*. *(V*.*) guyanensis*, *L*. *(V*.*) lainsoni*, *L*. *(V*.*) shawi*, a hybrid of *L*. *(V*.*) braziliensis* and *L*. *(V*.*) peruviana*, and *Leishmania (Leishmania) amazonensis*. Besides the present epidemiological observations, the current study provided the following findings: 1) A hybrid of *L*. *(V*.*) braziliensis* and *L*. *(V*.*) peruviana* is present outside the Department of Huanuco, the only place reported, 2) Many cases of CL due to *L*. *(V*.*) lainsoni*, an uncommon causative species in Peru, were observed, and 3) *L*. *(V*.*) shawi* is widely circulating in southern Amazonian areas in Peru.

## Author summary

Leishmaniasis, a neglected tropical disease (NTD) caused by the intracellular protozoa of the genus *Leishmania*, affects at least 12 million people in 96 countries. Peru is one of the most highly endemic countries for cutaneous leishmaniasis (CL), and our previous study identified *Leishmania (Viannia) braziliensis*, *L*. *(V*.*) peruviana*, and *L*. *(V*.*) guyanensis* in the tropical rainforest, in the Andean highlands, and in the northern and central rainforest areas, respectively, as the main CL-causative agents. In addition, distribution of *L*. *(V*.*) lainsoni*, *L*. *(V*.*) shawi*, a hybrid of *L*. *(V*.*) braziliensis* and *L*. *(V*.*) peruviana*, and *Leishmania (Leishmania) amazonensis* has been identified. Of these, one case each of *L*. *(V*.*) shawi* infection was reported from the Departments of Junin and Madre de Dios, while clinical cases due to the hybrid of *L*. *(V*.*) braziliensis* and *L*. *(V*.*) peruviana* were recorded only in the Department of Huanuco. To further elucidate the current geographic distribution of causative *Leishmania* species in Peru, a countrywide survey, including central to southern rainforest areas where little information on causative parasites is available, was performed based on the cytochrome *b* (*cyt* b) gene sequence and PCR-RFLP analysis of the mannose phosphate isomerase (*mpi*) gene by using FTA (Flinders Technology Associates) card-spotted samples and smear slides as DNA sources. In addition to current epidemiological observations, the current study revealed that 1) A hybrid of *L*. *(V*.*) braziliensis* and *L*. *(V*.*) peruviana* was identified, for the first time, outside the Department of Huanuco, 2) *L*. *(V*.*) lainsoni*, an uncommon CL-causative species in Peru, was predominantly found in the Department of Puno, where causative *Leishmania* species are not well-studied, and 3) New endemic foci of *L*. *(V*.*) shawi* were identified in central to southern rainforest areas of Peru.

## Introduction

Leishmaniasis, caused by an intracellular protozoa of the genus *Leishmania*, is a neglected tropical disease widely distributed worldwide, especially in tropical and subtropical areas, affecting at least 12 million people in 96 countries [1]. Approximately 20 *Leishmania* species belonging to the subgenera *Leishmania* (*Leishmania*), *Leishmania* (*Viannia*) and recently, *Leishmania (Mundinia)* are known to be pathogenic to humans and cause cutaneous, mucocutaneous and visceral disorders in infected individuals [1, 2]. Since infected parasite species are known to be the major determinant of clinical outcomes and may be associated with the response to treatments in leishmaniasis [1], identification of the infected parasite is important for appropriate treatment and prognosis.

Peru is one of the most highly endemic countries for cutaneous leishmaniasis (CL) [1]. Countrywide surveillance of leishmaniasis has been carried out, and the main causative parasites were identified as *L*. *(V*.*) peruviana*, *L*. *(V*.*) braziliensis*, and *L*. *(V*.*) guyanensis* in the Andean highlands, in the tropical rainforest, and in the northern to central rainforest areas, respectively [3–5]. Other than the three dominant species, distribution of *L*. *(V*.*) lainsoni* and *L*. *(L*.*) amazonensis* has been reported in lower-altitude rainforest areas [3–5]. In addition, a hybrid of *L*. *(V*.*) braziliensis* and *L*. *(V*.*) peruviana* was reported in the Department of Huanuco in 1995, and the current prevalence in the same areas was confirmed [6, 7]. Interestingly, CL due to the hybrid *Leishmania* has not been reported in other areas, although the infection seemed to be the most dominant in the Department of Huanuco [7]. On the other hand, two cases infected by *L*. *(V*.*) shawi* were newly reported in lowland rainforest areas in the Departments of Junin and Madre de Dios in 2010 [5]; since then, however, the infection by this species has not been reported in the country. Previous epidemiological studies have been conducted mainly in Andean areas facing the Pacific Ocean and central to northern rainforest areas, probably because of accessibility [3–5]. To obtain further information on prevalent *Leishmania* species in Peru, a countrywide survey, including central to southern rainforest areas, was performed based on the cytochrome *b* (*cyt* b) gene sequence and PCR-RFLP analysis of the mannose phosphate isomerase (*mpi*) gene by using FTA (Flinders Technology Associates) card-spotted samples and smear slides as DNA sources.

## Materials and methods

### Sample collection

Clinical samples were collected from patients suspected of having CL at 41 sites in 28 provinces of 13 departments in Peru during 2012 and 2017 (S1 Fig). Clinical samples spotted on FTA cards (Whatman, Newton Center, MA) and Giemsa-stained smears that were used for the diagnosis of CL were utilized in this study. To prepare FTA samples, tissue samples were taken by scraping the margins of active lesions of each patient, spotted onto an FTA Classic Card and stored at room temperature. Two-mm-diameter disks were punched out from each filter paper and washed twice with an FTA Purification Reagent (Whatman) and once with Tris-EDTA buffer. The disks were air-dried and directly subjected to PCR amplification. To extract DNA from Giemsa-stained smears obtained from skin lesions (ulcers and/or nodules) on CL patients, 50 μl of DNA extraction buffer [150 mM NaCl, 10 mM Tris-HCl (pH 8.0), 10 mM EDTA and 0.1% sodium dodecyl sulfate (SDS)] containing 100 μg/ml of proteinase K were spotted on each smear and mixed well. Detached tissue materials in the DNA extraction buffer were transferred to 1.5 ml tubes, incubated at 37˚C overnight, and heat-inactivated at 95˚C for 5 min. Each 0.5-μl portion was directly used as a template for PCR.

### Identification of *Leishmania* species

*Leishmania* species were identified by cytochrome *b* (*cyt* b) gene sequence analysis [5, 8]. PCR amplification with a pair of outer primers, L.cyt-AS (5'-GCGGAGAGRARGAAAAGGC-3') and L.cyt-AR (5'-CCACTCATAAATATACTATA-3'), was performed with 30 cycles of denaturation (95 ˚C, 1 min), annealing (55 ˚C, 1 min) and polymerization (72 ˚C, 1 min) using Ampdirect Plus reagent (Shimadzu Biotech, Tsukuba, Japan). Each 0.5-μl portion of the PCR product was reamplified with a pair of inner primers, L.cyt-S (5'-GGTGTAGGTTTTAGTYTAGG-3') and L.cyt-R (5'-CTACAATAAACAAATCATAATATRCAATT-3') under the same conditions described above. The products were cloned into the pGEM-T Easy Vector System (Promega, Madison, WI) and sequences were determined by the dideoxy chain termination method using a BigDye Terminator v3.1 Cycle Sequencing Kit (Applied Biosystems, Foster City, CA). The parasite species were identified based on their homology with *cyt* b gene sequences from *Leishmania* reference strains. Differentiation between *L*. *(V*.*) braziliensis* and *L*. *(V*.*) peruviana* was performed by a PCR- RFLP analysis of the mannose phosphate isomerase (*mpi*) gene using a restriction enzyme, *Vpa*K11BI (*Ava*II), as described previously [8].

### Phylogenetic analysis

The *Leishmania cyt* b gene sequences were aligned with CLUSTAL W software [9] and examined using the program MEGA (Molecular Evolutionary Genetics Analysis) version 6 [10]. A phylogenetic tree was constructed by the maximum likelihood (ML) method with the Hasegawa-Kishino-Yano (HKY) + G (Gamma distribution with 5 rate categories) model [10]. Branch support for the ML tree was calculated using the bootstrapping method with 1,000 replicates [10]. The best ML model for analysis was selected based on the lowest BIC score (Bayesian Information Criterion) in MEGA 6 [10]. The database for phylogenetic analyses consisted of *cyt* b gene sequences from 12 *Leishmania* species, *L*. *(L*.*) donovani* (GenBank accession number: AB095957), *L*. *(L*.*) infantum* (AB095958), *L*. *(L*.*) tropica* (AB095960), *L*. *(L*.*) major* (AB095961), *L*. *(L*.*) mexicana* (AB095963), *L*. *(L*.*) amazonensis* (AB095964), *L*. *(V*.*) braziliensis* (AB095967), *L*. *(V*.*) panamensis* (AB095968), *L*. *(V*.*) guyanensis* (AB095969), *L*. *(V*.*) naiffi* (AB433279), *L*. *(V*.*) lainsoni* (AB433280) and *L*. *(V*.*) shawi* (AB433281).

### Ethics statement

Clinical samples were collected by local physicians and well-trained laboratory technicians at health centers of the Ministry of Health, Peru. For routine parasitological diagnosis, scratching smear samples of skin lesions were taken from suspected leishmaniasis patients at health centers. In this study, only residual tissue materials were collected after the routine procedure to minimize the burden on patients. Signed consent was obtained from adult subjects and from children’s parents or guardians prior to the diagnostic procedures at each health center of the Ministry, providing information on the process of diagnosis and *Leishmania* species analysis, following the guidelines of the Ethics Committee of the Ministry. The subjects studied were volunteers in routine diagnosis/screening and treatment programs promoted by the Ministry. All routine laboratory examinations were carried out free of charge, and treatment with a specific drug, pentavalent antimony, was also offered free of charge at each health center. The study was approved by the ethics committee of the Graduate School of Veterinary Medicine, Hokkaido University (approval number: vet26-4) and Jichi Medical University (approval number: 17–080).

## Results

**Fig 1:**
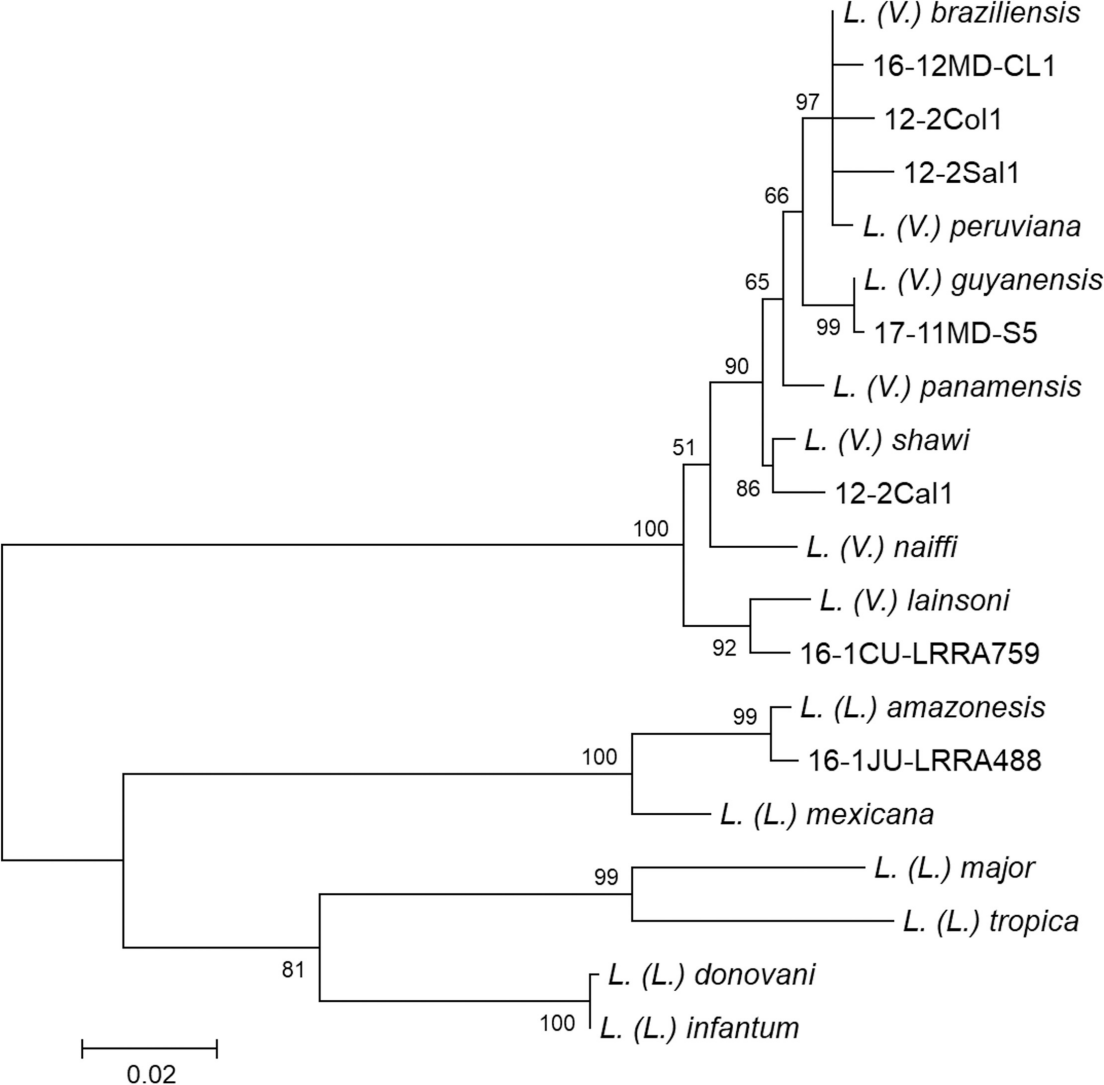
Phylogenetic tree of cytochrome *b* gene sequences among species. Leishmanial *cyt* b genes were amplified and sequenced from patients with cutaneous leishmaniasis, and a phylogenetic analysis was performed by the maximum likelihood method together with sequences from 13 *Leishmania* species. The scale bar represents 0.02% divergence. Bootstrap values are shown above or below branches. 16-12MD-CL1, 12-2Col1, 12-2Sal1, 17-11MD-S5, 12-2Cal1, 16-1CU-LRRA759 and 16-1JU-LRRA488 were sample names collected from the Departments of Madre de Dios, Cajamarca, Lambayeque, Madre de Dios, Cusco, Cusco and Junin, respectively.

**Fig 2:**
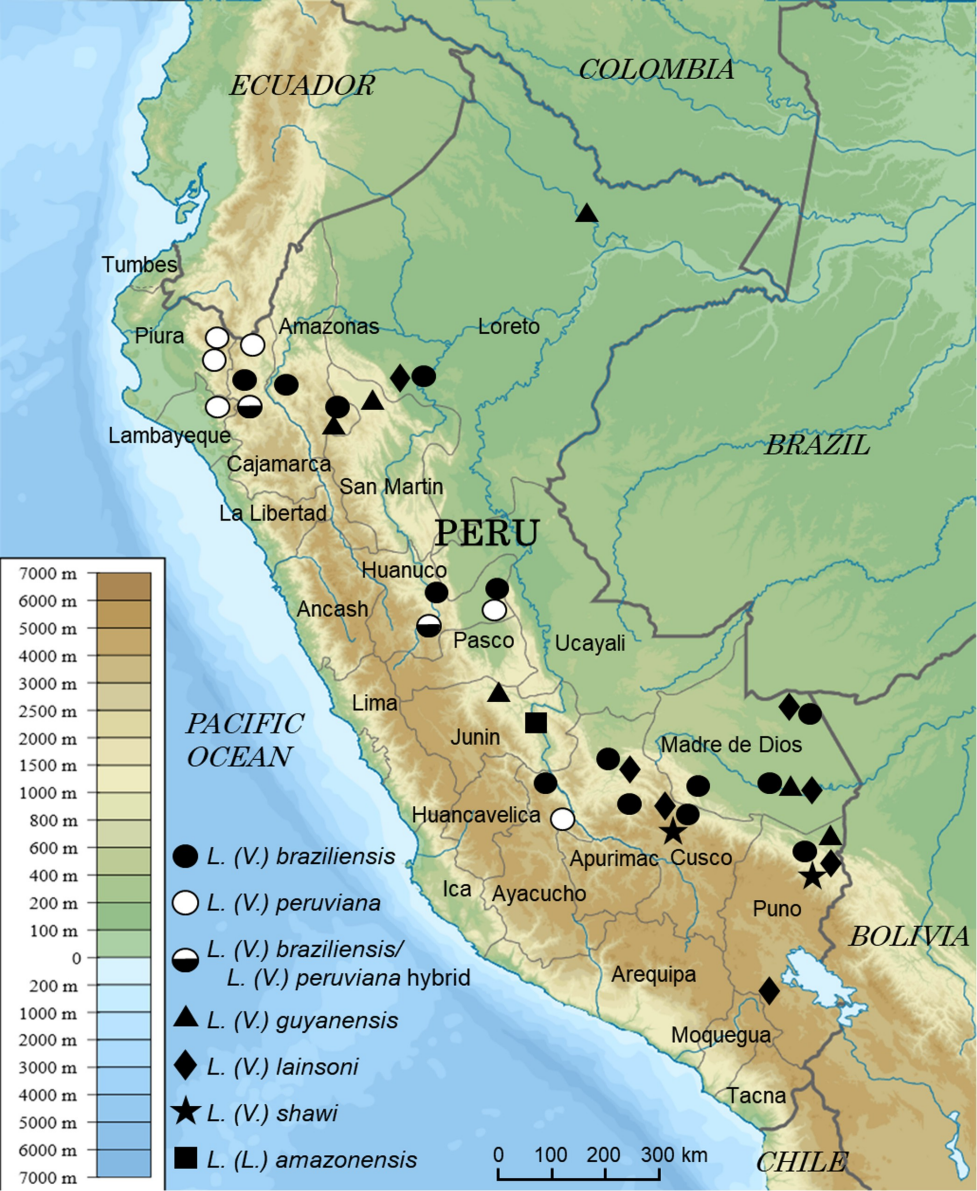
Geographic distribution of *Leishmania (Viannia) braziliensis*, *L*. *(V*.*) peruviana*, a hybrid of *Leishmania (V*.*) braziliensis* and *L*. *(V*.*) peruviana*, *L*. *(V*.*) guyanensis*, *L*. *(V*.*) lainsoni*, *L*. *(V*.*) shawi* and *Leishmania (Leishmania) amazonensis* by department in Peru. Each symbol represents the location where one or more specimens were collected. (Adapted from a map available at https://commons.wikimedia.org/wiki/File%3APeru_physical_map.svg).

A total of 262 clinical samples were collected from patients suspected of having CL in 28 provinces of 13 departments in Peru. Of these, 99 samples were collected on FTA cards, and 163 samples were Giemsa-stained smears. Parasite species were identified based on *cyt* b gene sequence analysis and phylogenetic analysis (Fig 1). Since *L*. *(V*.*) braziliensis* and *L*. *(V*.*) peruviana* were indistinguishable by *cyt* b gene analysis, the two species were further differentiated by PCR-RFLP analysis of the *mpi* gene [5, 7, 8]. The PCR amplification was repeated up to three times to obtain specific gene fragments. As the result, *Leishmania* DNAs were successfully amplified and species were identified in 83 (83.8%) of 99 FTA-spotted samples and 59 (36.2%) of 163 Giemsa-stained smear samples. The nucleotide sequence data reported in this paper will appear in the DDBJ, EMBL and GenBank databases under the accession numbers LC472411-LC472486 and LC472841- LC472880. The distribution of *Leishmania* species by department is presented in Table 1 and Fig 2. Among 142 samples identified, the most dominant species was *L*. *(V*.*) braziliensis* (47.2%), followed by *L*. *(V*.*) peruviana* (26.1%). Corresponding with previous studies [3–5], *L*. *(V*.*) braziliensis* was detected in lowlands, mainly in Amazonian areas, such as the Departments of Madre de Dios, Puno, Loreto, Huanuco, Cusco, Ayacucho, Amazonas, and Cajamarca, whereas *L*. *(V*.*) peruviana* distributed mostly in Andean highland areas such as the Departments of Lambayeque, Piura, Huanuco, Ayacucho, and Cajamarca (Table 1, Fig 2). A hybrid of *L*. *(V*.*) braziliensis* and *L*. *(V*.*) peruviana*, showing a hybrid RFLP pattern of the *mpi* gene (S2 Fig), was detected in the Department of Huanuco, where the hybrid parasite has been reported [6, 7] (Table 1, Fig 2). Unexpectedly, the hybrid parasite was also detected in four patients from an endemic area in the Department of Cajamarca, in which distribution of *L*. *(V*.*) braziliensis* and *L*. *(V*.*) peruviana* has been reported [3–5] (Table 1, Fig 2). This is the first report on the distribution of a hybrid of *L*. *(V*.*) braziliensis* and *L*. *(V*.*) peruviana* outside the Department of Huanuco. *Leishmania (V*.*) guyanensis*, a relatively dominant species in Peru, was detected in rainforest areas in Departments of Amazonas, Junin, Loreto, Madre de Dios, Puno, and San Martin, corresponding with previous observations [3–5] (Table 1, Fig 2). Infection by *L*. *(V*.*) lainsoni* is much less common in Peru; however, many cases of this infection were identified in the Department of Puno where causative species of CL have not been well-studied (Table 1, Fig 2). Two cases of *L*. *(V*.*) shawi* infection were reported in the Departments of Madre de Dios and Junin in 2010 [5], but no infections have been recorded since then. The present study identified *L*. *(V*.*) shawi* in the Departments of Cusco and Puno, suggesting that this species is widely distributed in southern Amazonian areas in Peru, although it is still less common (Table 1, Fig 2). A case of *L*. *(L*.*) amazonensis* infection was detected in the Department of Junin, where the infection has been reported previously [3].

**Table 1:** Distribution of *Leishmania* species by department in Peru.

**Department**	*Leishmania* species^1)^						
	Lb	Lp	Lb/Lp^2)^	Lg	Ll	Ls	La
Amazonas	2	0	0	2	0	0	0
Ayacucho	3	1	0	0	0	0	0
Cajamarca	1	1	4	0	0	0	0
Cusco	3	0	0	0	3	1	0
Huanuco	5	1	2	0	0	0	0
Junin	0	0	0	1	0	0	1
Lambayeque	0	24	0	0	0	0	0
Loreto	3	0	0	1	1	0	0
Madre de Dios	43	0	0	1	2	0	0
Piura		10	0	0	0	0	0
Puno	7	0	0	1	16	1	0
San Martin	0	0	0	1	0	0	0
Total	67	37	6	7	22	2	1

^1)^Lb, *L*. *(V*.*) braziliensis*; Lp, *L*. *(V*.*) peruviana*; Lg, *L*. *(V*.*) guyanensis*

Ll, *L*. *(V*.*) lainsoni*; Ls, *L*. *(V*.*) shawi*; Lm, *L*. *(L*.*) amazonensis*.

^2)^a hybrid of *L*. *(V*.*) braziliensis* and *L*. *(V*.*) peruviana*

In this study, all patients had typical ulcerative and/or nodular cutaneous lesions, and none had mucosal or mucocutaneous involvement. The number of cutaneous lesions per patient ranged from one to four, and the diameter of lesions ranged from 0.5 to 6cm. No marked characteristic differences in cutaneous lesions were observed among the causative *Leishmania* species identified.

## Discussion

Countrywide epidemiological studies of leishmaniasis have been conducted in Peru, and causative parasite species have been studied mainly in Andean areas and central to northern rainforest areas. To obtain further information on leishmaniasis widely endemic in Peru, CL-causing *Leishmania* species were investigated, especially focusing on central to southern rainforest areas where little information on causative parasite species is available, based on *cyt* b and *mpi* gene analyses. In terms of epidemiological observations, our study added the following findings: 1) A hybrid of *L*. *(V*.*) braziliensis* and *L*. *(V*.*) peruviana* is prevalent outside the Department of Huanuco, 2) Many CL cases in the Department of Puno were caused by *L*. *(V*.*) lainsoni*, which is an uncommon causative species in Peru, and 3) *L*. *(V*.*) shawi* is widely distributed in southern Amazonian areas in Peru.

In this study, samples spotted on FTA cards and smear slides used for parasitological diagnosis were applied as DNA sources. Use of FTA cards for direct sampling from patients’ lesions has several advantages such as a minimal risk of contamination, being less invasive, and ease of sample collection as reported previously [5, 11, 12]. When the cards were initially applied to sample collection for the epidemiological study on leishmaniasis, the detection efficacy of the parasite gene was relatively low (61.4%, 81/132 samples), probably because of a lack of information for optimal sampling procedures [5]. However, the sampling efficacy was improved in a subsequent study (75.8%, 125/165 samples) [12], and reached 83.8% (83/99 samples) in this study, indicating that the FTA card is a powerful tool for epidemiological studies if used properly. On the other hand, in Giemsa-stained smear samples, successful identification of parasite species was lower, with a PCR-positive ratio of only 36.2% (59/163 samples). A similar result was obtained in our previous study with a positive ratio of 42.7% (114/267 samples) when Giemsa-stained smears were applied as a DNA source [7]. As discussed previously, the inefficiency was probably due to the poor DNA condition in smear samples because it was lost and damaged during the routine processes of fixation by methanol, Giemsa staining, and removal of oil after parasitological diagnosis under a microscope [7].

Distribution of a hybrid of *L*. *(V*.*) braziliensis* and *L*. *(V*.*) peruviana* has been reported only in the Department of Huanuco, located in the mid-eastern region of the Peruvian Andes [3, 4, 7]. The present study identified CL cases due to the hybrid strain in an area of the Department of Cajamarca located in northern Peru. This is the first report of the prevalence of the hybrid strain outside the Department of Huanuco. The hybrid strain may have newly emerged in the Department of Cajamarca rather than originating from the Department of Huanuco, since the two endemic areas are more than 500 km apart and isolated by the central and eastern mountain ranges. Detailed molecular analysis such as population genetics using microsatellite markers may clarify the relationships between the hybrid strains. Further research on the prevalence of a hybrid of *L*. *(V*.*) braziliensis* and *L*. *(V*.*) peruviana* in other departments will be necessary. Importantly, the hybrid of *L*. *(V*.*) braziliensis* and *L*. *(V*.*) peruviana* was suggested to exacerbate the disease when compared to parental strains in an experimental animal model [13]. More precise clinical and epidemiological studies on individuals infected with the hybrid should be done to elucidate the relationships between the parasite and disease severity.

In this study, a high ratio of CL cases caused by *L*. *(V*.*) lainsoni* was identified in endemic areas of the Department of Puno, where CL-causative species are not well-studied. *L*. *(V*.*) lainsoni* is not a common causative species in Peru, and the infection was reported mainly in rainforest regions. The endemic areas in the Department of Puno may be unique and suitable areas to investigate the pathophysiology caused by *L*. *(V*.*) lainsoni*, drug efficacy against the infection, and its transmission mechanism including reservoirs and vectors.

*Leishmania (V*.*) shawi* was originally identified as a parasite of wild animals such as monkeys, sloths and procyonids in Amazonian Brazil [14]. Human infection has been recorded in rainforest areas of northern and northeastern Brazil [15, 16], and two CL cases caused by this species were reported in 2010 in lowland rainforest areas of the Departments of Junin and Madre de Dios in Peru; the first reports of *L*. *(V*.*) shawi* infection outside Brazil [5]. However, no infection by *L*. *(V*.*) shawi* has been reported in Peru since then. The present study identified two additional cases due to *L*. *(V*.*) shawi* infection in other areas, the Departments of Cusco and Puno, indicating that *L*. *(V*.*) shawi* is widely distributed in southern Amazonian areas of Peru. Interestingly, *L*. *(V*.*) shawi* infection occurs sporadically in Peru, and four cases from four geographically isolated areas have been reported. The parasite strain distributing in Peru may cause disease in particular populations such as immunocompromised people. Alternatively, sand fly species that transmit *L*. *(V*.*) shawi* may prefer to feed on animals rather than humans, resulting in a steady rate of infections in wild animals via sand fly bites and a lower risk of infection for humans. However, further epidemiological studies may find other endemic areas of *L*. *(V*.*) shawi* infection as observed with *L*. *(V*.*) lainsoni* in this study.

The present epidemiological study on leishmaniasis provided further insights into the distribution of *Leishmania* species in Peru, including an additional endemic area of a hybrid of *L*. *(V*.*) braziliensis* and *L*. *(V*.*) peruviana*, as well as new findings on the distribution of *L*. *(V*.*) lainsoni* and *L*. *(V*.*) shawi*. Further genetic analyses such as population genetics will be of help to reveal the origin of these parasites in each endemic area reported. In addition, it will be important to elucidate their transmission cycles, including reservoirs and vectors, to better understand and control leishmaniasis in Peru.

## Supporting information

S1 Fig Sample collection sites in Peru. Provinces of Utcubamba (1) and Rodriguez de Mendoza (2), Department of Amazonas; Provinces of Huanta (3) and La Mar (4), Department of Ayacucho; Provinces of San Ignacio (5) and Jaen (6), Department of Cajamarca; Provinces of La Convencion (7), Calca (8), and Paucartambo (9), Department of Cusco; Provinces of Leoncio Prado (10), Huanuco (11), and Puerto Inca (12), Department of Huanuco; Provinces of Chanchamayo (13) and Satipo (14), Department of Junin; Province of Lambayeque (15), Department of Lambayeque; Provinces of Maynas (16) and Alto Amazonas (17), Department of Loreto; Provinces of Tahuamanu (18), Tambopata (19), and Manu (20), Department of Madre de Dios; Provinces of Ayabaca (21) and Huancabamba (22), Department of Piura; Provinces of Carabaya (23), Sandia (24), and Puno (25), Department of Puno; Province of Rioja (26), Department of San Martin; Provinces of Coronel Portillo (27) and Atalaya (28), Department of Ucayali. (Adapted from a map available at http://english.freemap.jp/)
(TIF)

S2 Fig Direct sequence analysis showing a species-specific polymorphic site of Leishmania mannose phosphate isomerase (*mpi*) gene fragments. A. *L*. *(V*.*) braziliensis*, B. *L*. *(V*.*) peruviana*, C. Sample No. 12-2Chu2 from the Department of Huanuco, D. Sample No. 12-2Col2 from the Department of Cajamarca.
(TIF)
