# Supplementary figures and images for: Further insight into the geographic distribution of Leishmania species in Peru by cytochrome b and mannose phosphate isomerase gene analyses
Source: PLoS Negl Trop Dis. 2019 Jun 20;13(6):e0007496. doi: 10.1371/journal.pntd.0007496 (PMC6605678; doi:10.1371/journal.pntd.0007496)

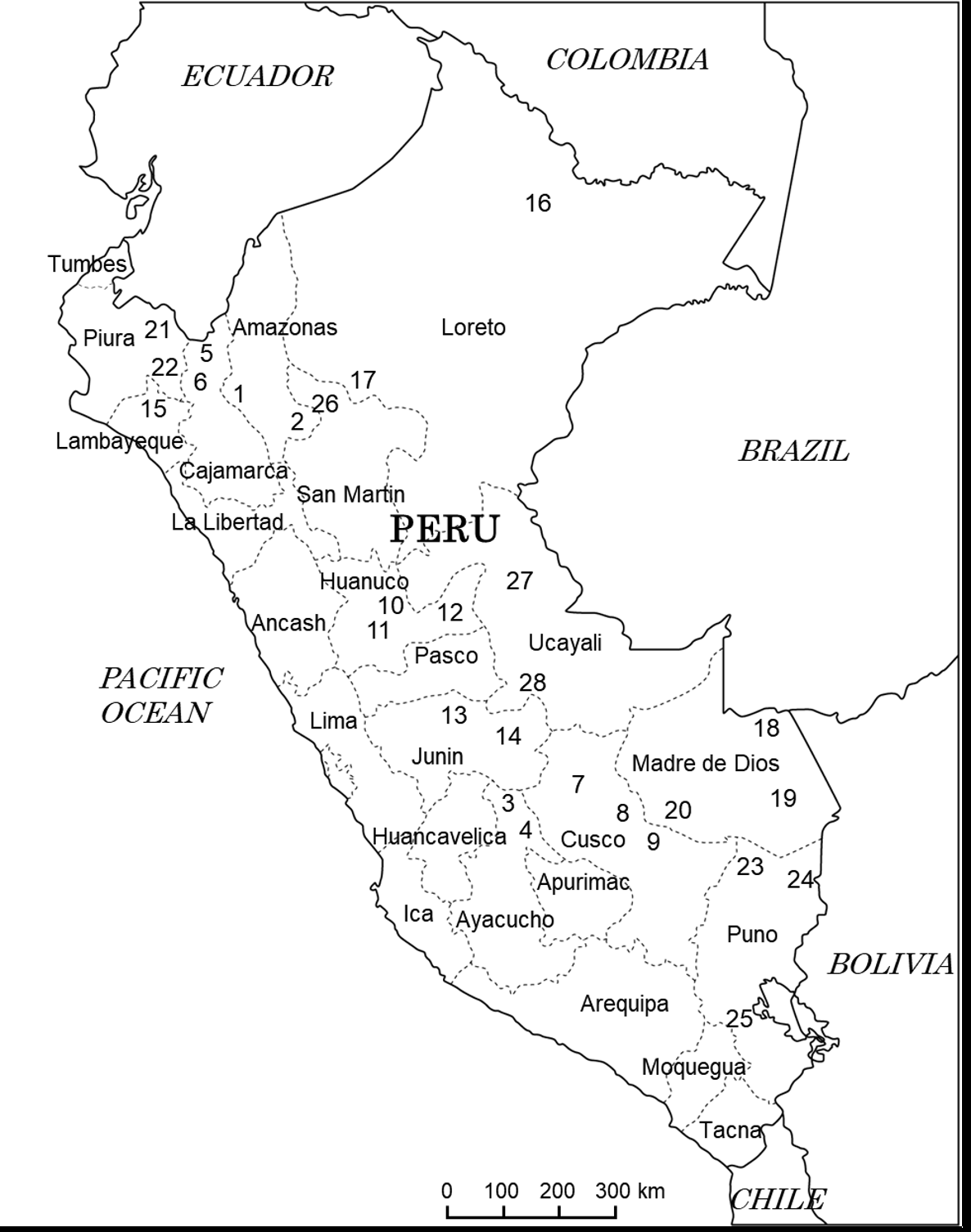

Supplement: S1 Fig — Provinces of Utcubamba (1) and Rodriguez de Mendoza (2), Department of Amazonas; Provinces of Huanta (3) and La Mar (4), Department of Ayacucho; Provinces of San Ignacio (5) and Jaen (6), Department of Cajamarca; Provinces of La Convencion (7), Calca (8), and Paucartambo (9), Department of Cusco; Provinces of Leoncio Prado (10), Huanuco (11), and Puerto Inca (12), Department of Huanuco; Provinces of Chanchamayo (13) and Satipo (14), Department of Junin; Province of Lambayeque (15), Department of Lambayeque; Provinces of Maynas (16) and Alto Amazonas (17), Department of Loreto; Provinces of Tahuamanu (18), Tambopata (19), and Manu (20), Department of Madre de Dios; Provinces of Ayabaca (21) and Huancabamba (22), Department of Piura; Provinces of Carabaya (23), Sandia (24), and Puno (25), Department of Puno; Province of Rioja (26), Department of San Martin; Provinces of Coronel Portillo (27) and Atalaya (28), Department of Ucayali. (Adapted from a map available at http://english.freemap.jp/) (TIF) [file pntd.0007496.s001.tif]

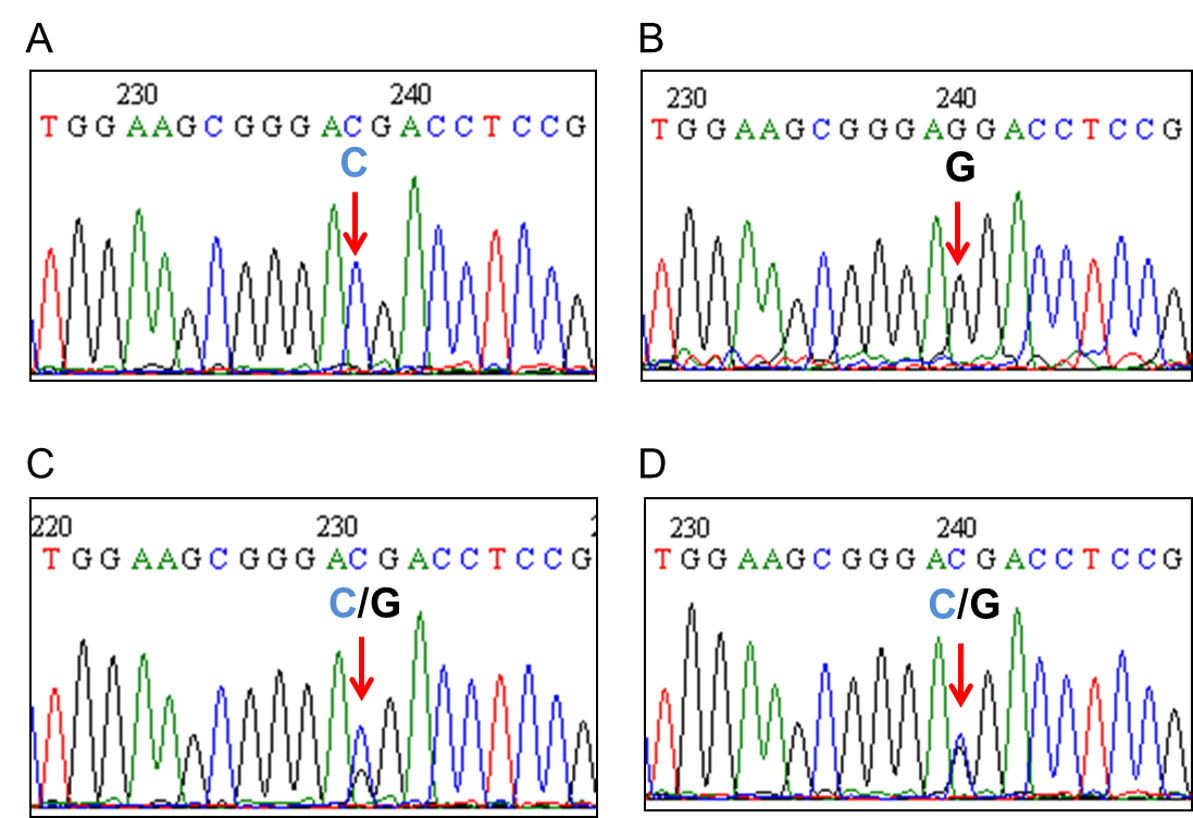

Supplement: S2 Fig — A. L. (V.) braziliensis, B. L. (V.) peruviana, C. Sample No. 12-2Chu2 from the Department of Huanuco, D. Sample No. 12-2Col2 from the Department of Cajamarca. (TIF) [file pntd.0007496.s002.tif]
